# Professional Freedom

**Published:** 1907-11-09

**Authors:** George W. F. Robbins

**Affiliations:** Secretary, Battersea General Hospital.


### EDITOR'S LETTER-BOX.

[Our Correspondents are reminded that prolixity is a great bar to publication, and that brevity of style and conciseness of statement greatly facilitate early insertion.]

### "PROFESSIONAL FREEDOM."

SIR,—I do not consider your comments in the issue of THE HOSPITAL of October 26 are quite fair. Your original article headed "Professional Freedom" dealt at great length with a variety of points in, as I endeavoured to prove, a somewhat intolerant spirit. When I reply almost *seriatim* to these many points, you not only decline to publish my letter, alleging its length, but endeavour to shelter yourself behind a statement made by those who were not at the moment attacked.

Our medical and surgical staff are *not* prohibited from using, nor forbidden to use, "vivisection" as a means of curing patients. They openly declare their own objection to, and abhorrence of, the practice; and no prohibition nor forbidding is necessary.

This Hospital was founded, indeed, as a protest against the vivisectionist system, and only those who are in sympathy with its principles are eligible for office.

Should you desire to go into the wisdom of the objections, we are quite ready to argue it with you, or with anyone else; and to prove that our staff produce better results without vivisection's misleading direction than any other hospital can produce under its faulty guidance.

I am yours faithfully,

GEORGE W. F. ROBBINS,

Secretary, Battersea General Hospital.

[We desire to afford Mr. Robbins every reasonable opportunity of presenting his case in what he considers its most effective form, and therefore we publish his letter *in extenso*. It will, however, be observed that he continues carefully to abstain from meeting the one and only issue raised in our article in reference to the Anti-Vivisection Hospital. That issue is contained in the statement that the medical officers of that hospital "are forbidden to make use of some kinds of treatment." To say, in reply to this, that "our staff are not prohibited from using 'vivisection' as a means of curing patients" may or may not be an intelligible proposition, but it certainly does not meet the charge, quoted above, and advanced by the Council of the Hospital Sunday Fund. With all respect to Mr. Robbins, we do not consider his views on the general questions raised in our article to be either of interest or value to our readers, and therefore we cannot accommodate them in our columns. His proposal to "argue" with us the therapeutic triumphs of the Anti-Vivisection Hospital would possibly have been more attractive had he addressed himself in a less elusive fashion to the simple and direct question on which he here claims to be heard.—ED. HOSPITAL.]

